# Cheese whey supports high riboflavin synthesis by the engineered strains of the flavinogenic yeast *Candida famata*

**DOI:** 10.1186/s12934-022-01888-0

**Published:** 2022-08-13

**Authors:** Justyna Ruchala, Yuliia A. Andreieva, Andriy O. Tsyrulnyk, Svitlana M. Sobchuk, Alicja Najdecka, Liu Wen, Yingqian Kang, Olena V. Dmytruk, Kostyantyn V. Dmytruk, Dariya V. Fedorovych, Andriy A. Sibirny

**Affiliations:** 1 Institute of Cell Biology, NAS of Ukraine, Drahomanov St, 14/16, Lviv, 79005 Ukraine; 2 University of Rzeszow, Zelwerowicza 4, 35-601 Rzeszow, Poland; 3 Guizhou Medical University, Guiyang, 550025 Guizhou China

**Keywords:** Riboflavin, Cheese whey, Yeast, *Candida famata*

## Abstract

**Background:**

Riboflavin is a precursor of FMN and FAD which act as coenzymes of numerous enzymes. Riboflavin is an important biotechnological commodity with annual market sales exceeding nine billion US dollars. It is used primarily as a component of feed premixes, a food colorant, a component of multivitamin mixtures and medicines. Currently, industrial riboflavin production uses the bacterium, *Bacillus subtilis,* and the filamentous fungus, *Ashbya gossypii*, and utilizes glucose and/or oils as carbon substrates.

**Results:**

We studied riboflavin biosynthesis in the flavinogenic yeast *Candida famata* that is a genetically stable riboflavin overproducer. Here it was found that the wild type *C. famata* is characterized by robust growth on lactose and cheese whey and the engineered strains also overproduce riboflavin on whey. The riboflavin synthesis on whey was close to that obtained on glucose. To further enhance riboflavin production on whey, the gene of the transcription activator *SEF1* was expressed under control of the lactose-induced promoter of the native β-galactosidase gene *LAC4*. These transformants produced elevated amounts of riboflavin on lactose and especially on whey. The strain with additional overexpression of gene *RIB6* involved in conversion of ribulose-5-phosphate to riboflavin precursor had the highest titer of accumulated riboflavin in flasks during cultivation on whey. Activation of riboflavin synthesis was also obtained after overexpression of the *GND1* gene that is involved in the synthesis of the riboflavin precursor ribulose-5-phosphate. The best engineered strains accumulated 2.5 g of riboflavin/L on whey supplemented only with (NH_4_)_2_SO_4_ during batch cultivation in bioreactor with high yield (more than 300 mg/g dry cell weight). The use of concentrated whey inhibited growth of wild-type and engineered strains of *C. famata*, so the mutants tolerant to concentrated whey were isolated.

**Conclusions:**

Our data show that the waste of dairy industry is a promising substrate for riboflavin production by *C. famata*. Possibilities for using the engineered strains of *C. famata* to produce high-value commodity (riboflavin) from whey are discussed.

**Supplementary Information:**

The online version contains supplementary material available at 10.1186/s12934-022-01888-0.

## Introduction

Riboflavin (vitamin B_2_) is an important water-soluble vitamin which serves as the biosynthetic precursor of flavin nucleotides ˗ flavin mononucleotide (FMN) and flavin adenine dinucleotide (FAD) that serve as coenzymes in many enzymatic reactions, mostly in oxidation and reduction [1–3]. Riboflavin has numerous applications with market value estimated to exceed 9 billion US dollars in 2021 [4]. It is primarily used as an animal feed additive, in food industry as a yellow colorant and in medicine as the component of multivitamin mixtures and as drug for treatment of some diseases [3, 5, 6]. Currently, riboflavin is produced biotechnologically using engineered strains of the bacterium, *Bacillus subtilis,* and the filamentous fungus, *Ashbya* (*Eremothecium*) *gossypii* [7, 8] on glucose or/and oil as carbon sources [9, 10]. Cheese whey is a nutrient rich by-product generated upon cheese production from milk, and constitutes a high-volume side stream in dairy industry. Biotechnological methods of its valorization are being sought. Cheese whey is also known to support riboflavin production in *A. gossypii* [11].

In current work, we studied the yeast *Candida famata* which naturally overproduces riboflavin under iron starvation [12, 13]. Riboflavin overproducing strains of *C. famata* in iron-sufficient media are known [14–16]. The *C. famata* dep8 strain isolated by classical selection, was used in the USA for industrial riboflavin production; however, due to high genetic instability and increased competition from low-cost producers, its production was shut down [1]. We independently constructed riboflavin-overproducing strains of *C. famata* by combination of methods of metabolic engineering and classical selection. A new *C. famata* strain was isolated that appeared to be very stable (non-reverting) and accumulated comparable concentrations of riboflavin to that of the strain dep8. Riboflavin synthesis in *C. famata* was studied mostly on glucose as the carbon source [15, 16].

Glucose is the primary carbon source used for cultivation of the industrial riboflavin producers. It is produced by enzymatic hydrolysis of starch. The price of riboflavin depends significantly on the price of the carbon substrate used. Profitability of riboflavin would be higher if a cheaper carbon source could be used provided that it maintains comparable product yield and yield with those more expensive substrate. The profit could be further is be increased if riboflavin could be produced from a waste stream. Cheese whey is a readily available abundant waste product. Whey contains lactose (around 5%) as the carbon source, and also proteins with a low amount of fat [17]. World production of bovine milk whey is estimated around 160 million tons per year [18]. Some of this whey is used for production of whey powder, whey proteins, powdered lactose, while another part is used as animal feed, fertilizer or discharged to sewage systems without treatment. Whey can also be anaerobically digested to methane as the major product [19, 20]. Biotechnological valorization of whey mostly involves bioethanol production using the lactose-utilizing yeasts, *Kluyveromyces marxianus* and *Kluyveromyces lactis* [21–23]. Other publications have also reported on the production of hydrogen, lactic acid and succinate from whey [19]. Only limited number of articles report on exploitation of whey as a substrate in synthesis of high-value substances, like squalene by *K. lactis* [24].

*C. famata* grows on lactose as a carbon source and displays β-galactosidase activity [25]. Recently, it was found that several strains of this species can overproduce riboflavin in a lactose-containing medium [26]. Here we report on the possible use of whey as carbon substrate for riboflavin production. Strains obtained previously as well as the newly constructed recombinant strains were studied for riboflavin production. We report that the engineered strains of *C. famata* overproduce riboflavin in whey-containing media. Altogether our data support the feasibility of using cheese whey, an abundant waste of dairy industry, in synthesis of a high-value added product, riboflavin.

## Materials and methods

### Growth conditions

Cultivation of *C. famata* strains in liquid media was carried out in the shakers (200 rpm) at 30 °C in 10 mL of liquid media in 100 mL Erlenmeyer flasks for 96 h. The following cultivation media were used: YPD (0.5% yeast extract, 1% peptone and 2% glucose); YNB (0.17% YNB, 0.3% ammonium sulfate) with 0.2% of yeast extract and 5% glucose; YNB in concentration 0.17% with 0.2% of yeast extract and 5% lactose; cheese whey with 5% of lactose and 0.3% (NH_4_)_2_SO_4_. Cheese whey was obtained from Mlekowita Company (Poland). Deproteinization was carried out by heat treatment (121 °C, 15 min) of the acidified (pH 5) cheese whey. The precipitates as a cheese-like sediment were removed by centrifugation at 4 °C and 8000×*g* for 5 min.

A preculture of the BRPI/RIB6 incubated with shaking 220 rpm at 28 °C for 24 h on YPD was used to inoculate the starting volume (600 ml of batch medium) of the bioreactors to a starting biomass 1 g DCW/L. Fermentations were carried out in 1.3-Liter total volume bioreactors (BioFlo 115, New Brunswick). Fermentation temperature was controlled at 28 °C, pH was controlled at 5.5 ± 0.5 with addition of 10% sodium hydroxide, and the dissolved-oxygen concentration was maintained above 50% saturation by controlling agitation speed between 200 and 500 rpm, whereas the airflow was kept constant at 1 L/min. The batch medium contained whey supplemented with ammonium sulfate 3 g/L.

### Plasmids and strains

Recombinant *C. famata* strains used throughout this work (Additional file 1: Table S1) have been described previously [15, 16, 26–29]. New recombinant strains were constructed by introduction of plasmids pNTC/pLAC4_cf-SEF1_cf (Additional file 1: Fig. S1A) [25] with expression cassette pLAC4-SEF1 into strains BRP/RFE1, BRPI to create BRP/RFE1/pLAC4-SEF1, BRPI/pLAC4-SEF1 (Table 1)*.*

**Table 1:** Riboflavin production of *C. famata* strains grown in YNB with different source of carbon or whey on 72 h *

Strains	Source of carbon	Biomass, g/L	Riboflavin	
			mg/L	mg/g DCW
AF-4	Glucose	4.32 ± 0.17	325.00 ± 13.00	75.26 ± 2.63
	Lactose	4.00 ± 0.16	152.50 ± 6.15	38.86 ± 1.36
	Whey	13.6 ± 0.65	498.00 ± 23.90	37.59 ± 1.80
BRP	Glucose	3.93 ± 0.14	366.00 ± 14.64	93.12 ± 3.26
	Lactose	3.77 ± 0.13	252.80 ± 9.18	67.05 ± 2.35
	Whey	9.63 ± 0.46	890.09 ± 44.14	92.43 ± 4.23
BRPI	Glucose	3.04 ± 0.12	822.50 ± 32.90	237.3 ± 8.69
	Lactose	3.21 ± 0.13	310.00 ± 12.40	96.57 ± 3.86
	Whey	9.88 ± 0.44	930.00 ± 46.50	94.13 ± 3.77
BRPI/RIB6	Glucose	3.31 ± 0.13	903.75 ± 35.15	273.20 ± 13.66
	Lactose	3.41 ± 0.15	310.40 ± 13.5	91.02 ± 4.55
	Whey	9.95 ± 0.39	1450.20 ± 72.51	145.75 ± 6.56
VKMY-9	Glucose	4.17 ± 0.17	2.75 ± 0.10	0.66 ± 0.03
	Lactose	3.42 ± 0.14	0.45 ± 0.02	0.13 ± 0.01
	Whey	9.67 ± 0.41	12.10 ± 0.60	1.25 ± 0.06

^*^YNB supplemented with of yeast extract (0.2%), 5% glucose, 5% lactose or whey

(5% of lactose) and ammonium sulfate (0.3%)

Obtaining of the strains with an overexpression of 6-phosphogluconate dehydrogenase was carried out by construction of corresponding plasmid. Gene *GND1* was PCR-amplified from genomic DNA of the wild-type strain *C. famata* VKMY-9 using pairs of primers Ko1054 (GCA CTG CAG GCG GCC GCA TGT CTG CTC CAA CGT ATG TAT TCT TC) / Ko1055 (GCA CTG CAG CTA AGC ATC GTA AGT AGA GGC AGA AAC). Fragment of *GND1* was treated with BamHI and NotI and cloned into the corresponding sites of pUC57_prTEF1*Cf*_trTEF1*Dh*_Ble_Sa – an expression vector containing the *C. famata TEF1* promoter, *D. hansenii TEF1* terminator, and a selective marker conferring resistance to phleomycin – gene *ble*, created previously on the base of pUC (Fermentas, Vilnius, Lithuania).

The constructed plasmid was designated as pGND1 (Additional file 1: Fig. S2A). The plasmid pGND1 was linearized at the site AhdI, and used for transformation of *C. famata* strains: L2, AF-4 and BRP as previously described [12]. Selected strains were named L2/pTEF1-GND1, AF-4/pTEF1-GND1 and BRP/pTEF1-GND1.

The transformants were selected on a YPD agar medium, containing nourseothricin 20 mg/L or phleomycin 30 mg/L, after 4–5 days of incubation. The selected transformants were stabilized by alternating cultivation on a non-selective followed by selective media. After stabilization, the selected strains were confirmed by PCR using pair of primers Ko1068 (TTT GGG CCC TAT ATA GAG ACA ATA AGA CCA G) /OL23 (AGG TTG AAG TGG GAA TTG CAT C) for BRP/RFE1/pLAC4-SEF1 and BRPI/pLAC4-SEF1 strains (Additional file 1: Fig. S1B) and pair of primers Ko1056 (CGG GGT ACC AAA TTG ACT GGT CTG AAA TAA TAG) /Ko1059 (GTT CAA TAA AAG CAT CAA CTG G) for strains (Additional file 1: Fig. S2B).

*Escherichia coli* strain DH5α (Φ80d*lac*ZΔM15, *rec*A1, *end*A1, *gyr*A96, *thi*-1, *hsd*R17(r-K m + K), *sup*E44, *rel*A1, *deo*R, Δ(*lacZYA-argF*) U169) was used in experiments that required a bacterial host. DH5α was grown at 37 °C in LB medium. Transformed *E. coli* cells were maintained in rich medium containing 100 mg/L of ampicillin.

### Assays

Cell biomass of yeast was determined with a Helios Gamma spectrophotometer (OD, 590 nm; cuvette, 10 mm) turbidimetrically with gravimetric calibration. Flavin production was analyzed by measuring fluorescence (Turner Quantech FM 109,510–33 fluorometer, excitation maximum = 440 nm, emission maximum = 535 nm).

### Quantitative Real-Time PCR

Expression of the *SEF1* and *GND1* genes was confirmed by qRT-PCR. Total RNA was extracted from yeast cells using the GeneMATRIX Universal RNA Purification Kit with DNAseI (EURx Ltd., Gdansk, Poland). The qRT-PCR was performed by 7500 Fast Real-Time PCR System (The Applied Biosystems, USA) with SG OneStep qRT-PCR kit (EURx Ltd., Gdansk, Poland) using corresponding pairs of primer LAC4_qFCf (GGA CCA GGT GAA AGT TAT AG) / LAC4_qRCf (CTA CCT TCC ATT GTA ACA TAA AG), GND1_Cf_f (ACC CAT TCT TCA ACG ATG CTA)/GND1_Cf_r (CAC TTG GAA AGT GTG GGC) and ACT1f (TAA GTG TGA TGT CGA TGT CAG)/ACT1r (TTT GAG ATC CAC ATT TGT TGG AA), RNA as a template and ROX reference passive dye following manufacturer’s instructions as previously described [28].

### Statistical analysis

All the experimental data shown in this manuscript were collected from three independent samples to ensure reproducibility of the trends and relationships observed in the cultures. Each error bar indicates the standard deviation (SD) from the mean obtained from triplicate samples. The 5% significance level was used in the statistical analyses.

## Results

### Riboflavin production by *C. famata* strains grown in different media

Previously, we found that *C. famata* is characterized by robust growth on lactose and riboflavin overproducing strains of this species can accumulate comparable amounts of this vitamin on both glucose and lactose [25, 26]. As whey contains lactose (near 5%) as the sole sugar, we decided to study riboflavin synthesis on this waste product by riboflavin overproducing strains we constructed earlier. Growth and riboflavin synthesis were studied in synthetic YNB media with glucose and lactose (both in concentrations 5%) as well as on whey supplemented with a nitrogen source (ammonium sulfate). The following strains were studied: AF-4 (riboflavin overproducer isolated by conventional mutagenesis and classical selection), BRP (isolated from AF-4 by introduction of additional copies of *SEF1, RIB1* and *RIB7* genes) and the wild-type strain VKM Y-9 [15] (Additional file 1: Table S1). It was found that whey supports robust growth of all these strains with riboflavin accumulation comparable to that on glucose and lactose (Table 1). The biomass levels by the analyzed strains were similar in glucose and lactose media whereas on whey with nitrogen source biomass accumulation was significantly (2.3–3.6-fold) higher than that in YNB with glucose or lactose (Table 1). The tested strains showed 1.4–2.9-fold higher riboflavin production and riboflavin yield (mg of riboflavin per g of DCW) in YNB with glucose as compared to that on lactose. Despite highest titer of riboflavin produced by BRP in whey reaching 890 mg/L, the riboflavin yield was close to that in YNB with glucose. Other strain AF-4 was characterized by increase in riboflavin production on whey relative to YNB/glucose and especially relative to YNB/lactose media; similarly, the riboflavin yield was the lowest in YNB medium with lactose (Table 1). Highest riboflavin synthesis on whey was produced by the strain BRPI/RIB6 with activated purine nucleotide synthesis de novo and overexpressed genes *SEF1, RIB1, RIB7* and *RIB6* [28, 29], where riboflavin level on whey supplemented with ammonium sulfate in shake flasks reached 1,45 g/L and yield of riboflavin synthesis was 145 mg of riboflavin/g dry cell weight (Table 1).

**Fig. 1:**
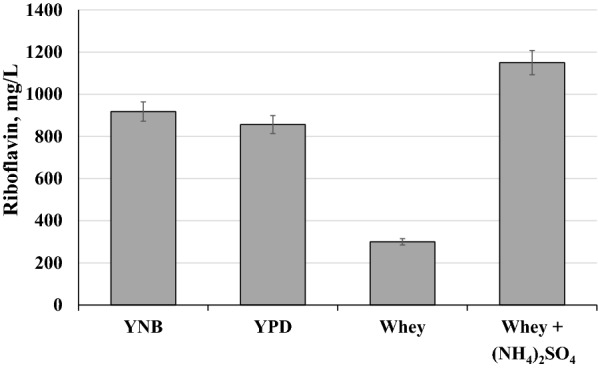
Riboflavin production by C. famata strain BRP/RFE1 grown in YNB, YPD, whey and whey supplemented with ammonium sulfate (0.3%) media during 72 hours. Error bars indicate standard deviations calculated from three independent experiments

Next experiment shows data on riboflavin synthesis by the strain BRP/RFE1 of *C. famata* with overexpression of the homolog of the mammalian riboflavin efflux protein, named as riboflavin excretase (*RFE1*) in the background of strain BRP with overexpression of genes *SEF1, RIB1* and *RIB7* [27]. It was found that whey without nitrogen source supported active riboflavin production though riboflavin titer was below that accumulated in YNB/glucose or YPD media. Addition of ammonium sulfate 3.8-fold activated riboflavin synthesis on whey, reaching 1150 mg/L (Fig. 1). Riboflavin production was similar in YNB and YPD media (Fig. 1).

### Riboflavin production by recombinant strains *C. famata* with expressed transcription activator *SEF1* under control of *LAC4* promoter

Recently, we showed that the overexpression of own transcription activator *SEF1* gene under control of promoter of the homologous *LAC4* gene encoding β-galactosidase in AF-4 and BRP strains led to increase in riboflavin production on media with lactose and whey [26]. In current study, we showed that *LAC4* expression in AF-4 is indeed activated by lactose or whey for 1.7- or 1.8-fold as compared to expression in glucose medium (Additional file 1: Table S2). Therefore, we suggested that expression of *SEF1* gene under control of *LAC4* promotor could be used for activation of riboflavin synthesis on whey. As parental strains, BRP/RFE1 with overexpression of *RFE1* gene coding for riboflavin excretase [27] and BRPI with activation of purine nucleotide synthesis de novo [28] were used. Resulted strains were named BRP/RFE1/pLAC4-SEF1 and BRPI/pLAC4-SEF1 (Additional file 1: Table S1).

**Fig. 2:**
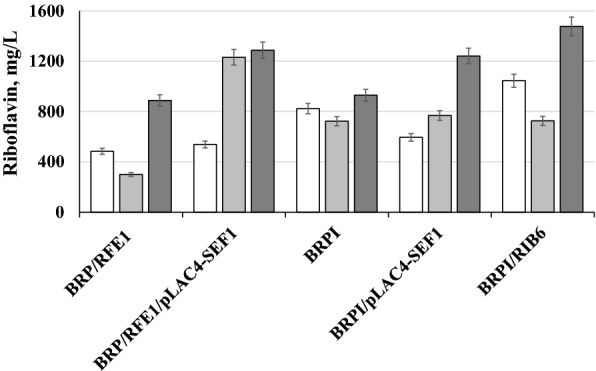
Riboflavin production by *C*. *famata *strains BRP/RFE1, BRP/RFE1/pLAC4-SEF1, BRPI, BRPI/pLAC4-SEF1 and BRPI/ 6 during growth on YNB with 0.2% yeast, 5% glucose (open), 5% lactose (grey) and whey supplemented with ammonium sulfate (dark grey) at 96 hours. Error bars indicate standard deviations calculated from three independent experiments

Riboflavin synthesis of the BRP/RFE1/pLAC4-SEF1 and BRPI/pLAC4-SEF1 in YNB-glucose medium did not differ from that in the parental strains BRP/RFE1 and BRPI, respectively (Fig. 2). Riboflavin production by strains BRPI, BRPI/pLAC4-SEF1, BRPI/RIB6 was very similar on YNB with lactose, varying from 723 to 769 mg/L (Fig. 2). At the same time, riboflavin production of the BRP/RFE1/pLAC4-SEF1 on YNB with lactose was characterized by 4.1-fold increase as compared to the parental strain BRP/RFE1 (Fig. 2). The strains BRP/RFE1/pLAC4-SEF1 and BRPI/pLAC4-SEF1 showed 1.5- and 1.3-fold increase in riboflavin production on whey as compared to that by the corresponding parental strains, accumulating 1288 and 1242 mg/L, respectively (Fig. 2). Obtained data confirm that lactose-inducible expression of *SEF1* increases riboflavin production in the lactose containing media, including whey.

### Riboflavin production on whey by the novel constructed *C. famata* overproducers

The highest riboflavin production during flask cultivation on whey (1477 mg/L) was observed in strain BRPI/RIB6 which contains overexpression of structural genes of riboflavin synthesis *RIB1, RIB6, RIB7*, gene encoding of positive regulator of riboflavin biosynthesis *SEF1* and engineered genes involved in purine nucleotide synthesis de novo *PRS3m* and *ADE4m* [29].

It is remarkable, that riboflavin accumulation in whey supplemented with only nitrogen source, was higher than that in semisynthetic media with 5% glucose or lactose supplemented with yeast extract. Apparently, whey contains all necessary elements for yeast cultivation with exception of nitrogen source which limits growth and should be added.

**Fig. 3:**
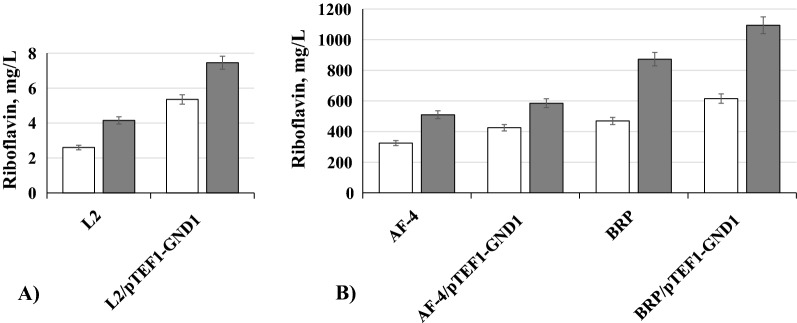
Riboflavin production by *C*. *famata* strains L2, L2/pTEF1-GND1 (**A**), AF-4, AF-4/pTEF1-GND1, BRP and BRP/pTEF1-GND (**B**) on 90 hours of growth in YNB medium with 5% glucose (open) and on whey supplemented with ammonium sulfate (dark grey). Error bars indicate standard deviations calculated from three independent experiments

Here, we also overexpressed gene *GND1* coding for 6-phosphogluconate dehydrogenase which converts 6-phosphogluconate to ribulose-5-phosphate, the aliphatic precursor of riboflavin [1] For this, gene *GND1* was expressed under control of strong constitutive promoter *TEF1*. The isolated transformants as expected, overexpressed *GND1* gene (Additional file 1: Table S2) and overproduced riboflavin in YNB medium with glucose and on whey supplemented with ammonium sulfate (Fig. 3). It is interesting to note that all strains produce more riboflavin on whey relative to that on YNB/ glucose. L2/pTEF1-GND1 showed twofold increase of riboflavin production on YNB with glucose as well as on whey relative to that of L2. Despite significant increase of *GND1* expression, strain AF-4/pTEF1-GND1 produced only 15% more riboflavin on whey than that the parental strain AF-4. At the same time, riboflavin production by AF-4/pTEF1-GND1 in YNB medium with 5% glucose showed 1.3-fold increase as compared to the parental strain (Fig. 3). Strain BRP/pTEF1-GND1 accumulated 1.3-fold more riboflavin in YNB/glucose and whey as compared to BRP. The highest titer of riboflavin was produced by BRP/pTEF1-GND1 on whey reaching 1094 mg/L (Fig. 3).

### Bioreactor cultivation

Among all tested strains, BRPI/RIB6 [29] with additional overexpression of gene *RIB6* involved in conversion of ribulose-5-phosphate to riboflavin precursor accumulated the highest titer of riboflavin in flasks during cultivation on whey, reaching 1.48 g/L (Fig. 3). Strain BRPI/RIB6 was cultivated in bioreactor during batch mode on whey with addition of ammonium sulfate. It was found that strain accumulated 2.5 g of riboflavin/L after 212 h of cultivation. Yield of riboflavin synthesis appeared to be as high as 308 mg of riboflavin/g of dry biomass.

### Riboflavin production by strain BRP and mutants of *C. famata*, grown on concentrated whey

**Fig. 4:**
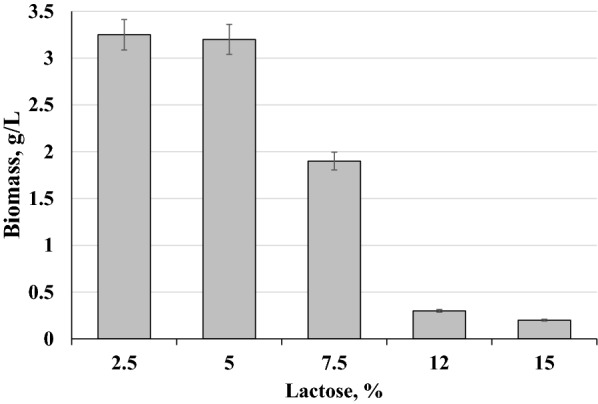
Growth of *C. famata *strains BRP on whey with different concentration of lactose at 96 hours. Error bars indicate standard deviations calculated from three independent experiments

**Fig. 5:**
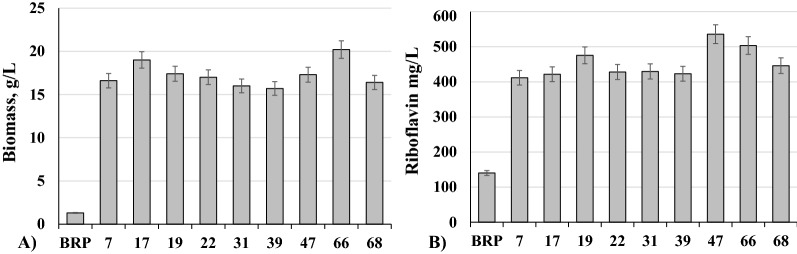
Growth **(A)** and riboflavin production **(B)** by strain BRP and mutants of C. famata, grown on whey with 12% of lactose with ammonium sulfate at 96 hours. Error bars indicate standard deviations calculated from three independent experiments

Concentrated whey is produced to reduce transportation costs. We have found that such concentrated whey with lactose concentration around 10—15% strongly inhibited growth of *C. famata* (Fig. 4). Inhibition was not caused by lactose as 10—15% lactose used as sole carbon source in YNB medium did not inhibit yeast growth (not shown). The substance of concentrated whey which inhibits yeast growth remains unknown. The UV-induced mutants with robust growth on concentrated (10–12%) whey have been isolated which also showed satisfactory accumulation of riboflavin during flask cultivation (Fig. 5). Still riboflavin production by the isolated mutants on concentrated whey containing 12% lactose was lower than that by parental strain BRP on the standard whey with 5% lactose (Table 1, Fig. 5).

Isolated mutants capable of growth on concentrated whey with 12% lactose could not grow on concentrated whey with 15% lactose and our repeated attempts to isolate such mutants failed. Strains capable of growth on concentrated whey are of great interest as transportation of such concentrated whey has significant cost saving.

## Discussion

Riboflavin overproducing microorganisms as a rule are cultivated in media with glucose and/or oil [1–3, 7–10]. Cost of the final product, riboflavin, strongly depends on the costs of the carbon substrate, therefore substitution of glucose or oil for cheaper substrate and especially for waste product like cheese whey, could be important for profitability of riboflavin production. Information on microbial riboflavin production on whey has been known for many years [11, 30, 31] however, only *A. gossypii* accumulates significant amounts of riboflavin on whey supplemented with wheat mill bran which is rich in various minerals, pentosans, starch, total sugar, sucrose, and reducing sugars [32]. In such medium, during bioreactor cultivation, concentration of riboflavin reached 1.2 g/L [11]. Data obtained in the current work show that the abundant waste of dairy industry, whey, could be used as effective substrate for riboflavin production using riboflavin-overproducing strains of the lactose-utilizing yeast *C. famata*. Riboflavin overproduction on whey could be especially interesting for countries with developed dairy industry like USA, Netherlands, Germany, Ireland, Poland and others. Maximal concentration of riboflavin (2.5 g/L) obtained in bioreactor cultivated on whey supplemented only with mineral nitrogen source exceeds levels achieved by other microorganisms. Apparently, whey contains all other macro- and microelements necessary for yeast growth and riboflavin oversynthesis. Yield of riboflavin synthesis on whey is quite satisfactory, similar to that on glucose and even higher in the most cases. Especially high yield of riboflavin synthesis (more than 300 mg of riboflavin per g dry weight) was found during BRPI/RIB6 strain cultivation on whey in bioreactor. Such yield is rarely achieved during cultivation in glucose media. We plan to study riboflavin accumulation on whey using fed-batch cultivation conditions which could result in higher biomass and riboflavin titers on whey. Some molecular approaches could be applied including cloning and overexpression of the *LAC4* and *LAC12* genes coding for β-galactosidase and β-galactoside permease, respectively. Overexpression of the mentioned genes could further improve lactose utilization and hence, riboflavin production on whey. We also plan to introduce pLAC4-SEF1 construct in the best currently available BRPI/RIB6 riboflavin producer to activate riboflavin production on whey. Identification of the toxic component(s) in the concentrated whey and isolation of the tolerant strains with higher riboflavin yield are also among our goals.

Especially interesting are our data on riboflavin overproduction due to overexpression of *GND1* gene on whey (Fig. 3). Gene *GND1* encodes 6-phosphogluconate dehydrogenase involved in oxidative branch of pentose phosphate pathway responsible for synthesis of ribulose-5-phosphate, which serves as aliphatic riboflavin precursor [1]. Till now, the role of 6-phosphogluconate dehydrogenase in yeasts and filamentous fungi was not studied whereas overexpression of the corresponding *gnd* gene from *Corynebacterium glutamicum* was found to be important for riboflavin oversynthesis in bacteria *B. subtilis* and *Escherichia coli* [33, 34]. Such overexpression apparently leads to increase in intracellular pool of ribulose-5-phosphate, the precursor of riboflavin. Increase in concentration of this metabolite due to deletion of gene coding for ribulose-5-phosphate epimerase in Bacillus subtilis also enhanced riboflavin production [35]. Analysis of the ribulose-5-phosphate pool in the constructed here *C. famata* strain with overexpression of *GND1* gene would be important for better understanding mechanisms involved in riboflavin overproduction in yeast. Role of glucose-6-phosphate dehydrogenase and 6-phosphogluconolactonase in riboflavin synthesis in *C. famata* is also interesting to study.

Thus, whey could be used as a promising substrate for the development of technology for industrial production of vitamin B_2_. Accumulated till now data could be considered as proof of concept in favor of such technology; however, its profitability should be carefully considered. Costs of whey transportation, yeast cultivation and riboflavin downstream processing should be evaluated.

## Conclusions

The ability of the engineered strains of the yeast *C. famata* to overproduce riboflavin on whey was established. The abundant waste of milk industry, whey, could be used as effective substrate for riboflavin production using riboflavin-overproducing strains of the lactose-utilizing yeast *C. famata*. Maximal riboflavin accumulation during bioreactor cultivation on whey supplemented with ammonium sulfate reached 2.5 g/L with yield exceeded 300 mg/g dry weight. Thus, the milk industry waste, whey, could be effectively converted to vitamin B_2_.

## Supplementary Information

**Additional file 1: Table S1.** *C. famata* yeast strains used in present study. **Table S2.** Relative expression levels of *LAC4* and *GND1*genes versus the recipient strains. **Figure S1.** Linear scheme of plasmid pNTC/pLAC4_cf-SEF1_cf and PCR verification of the corresponding transformants. **Figure S2.** Linear scheme of plasmid pGND1 and PCR verification of the corresponding transformants

## Data Availability

All data generated or analyzed during this study are included in this article and its additional file. If additional information is needed, please contact the corresponding author.
