# Supplementary material for: Cheese whey supports high riboflavin synthesis by the engineered strains of the flavinogenic yeast Candida famata
Source: Microb Cell Fact. 2022 Aug 13;21:161. doi: 10.1186/s12934-022-01888-0 (PMC9375410; doi:10.1186/s12934-022-01888-0)
Supplement: Supplementary file 1 — Additional file 1: Table S1. C. famata yeast strains used in present study. Table S2. Relative expression levels of LAC4 and GND1genes versus the recipient strains. Figure S1. Linear scheme of plasmid pNTC/pLAC4_cf-SEF1_cf and PCR verification of the corresponding transformants. Figure S2. Linear scheme of plasmid pGND1 and PCR verification of the corresponding transformants [file 12934_2022_1888_MOESM1_ESM.docx]

Additional file 1: Table S1. *C. famata* yeast strains used in present study. Table S2. Relative expression levels of *LAC4* and *GND1*genes versus the recipient strains. Figure S1. Linear scheme of plasmid pNTC/pLAC4_cf-SEF1_cf and PCR verification of the corresponding transformants. Figure S2. Linear scheme of plasmid pGND1 and PCR verification of the corresponding transformants.

Additional file 1 **Table 1**. *C. famata* yeast strains used in present study

| **Strain** | **Description** | **Reference** |
| --- | --- | --- |
| VKMY-9 | Wild type | All-Russian Collection of Microorganisms, Pushchino, Russia |
| L2 | Leucine auxotroph L20105 (*leu2*) | [12] |
| AF-4 | *C. famata* riboflavin overproducer isolated by conventional mutagenesis and classical selection | [15] |
| BRP | AF-4/SEF1/RIB1/RIB7 (designated as BRP from the Best Riboﬂavin Producer). AF-4 with introduction of additional copies of *SEF1, RIB1* and *RIB7* genes | [16] |
| BRP/RFE1 | BRP with introduction *RFE1* gene under control of *TEF1* promoter; | [27] |
| AF-4/pLAC4-SEF1 | AF-4 with overexpression of *SEF1* gene under control of lactose-inducible promoter *LAC4* gene | [26] |
| BRP/pLAC4-SEF1 | BRP with overexpression of *SEF1* gene under control of lactose-inducible promoter *LAC4* gene | [26] |
| BRP/RFE1/pLAC4-SEF1 | BRP/RFE1 with introduction of *SEF1* gene under control of lactose-inducible promoter *LAC4* gene | Present study |
| BRPI | BRP/PRS3m/ADE4m (designated as BRPI from the Best Riboﬂavin Producer Improved) with activation of modified genes *PRS3* and *ADE4* of purine nucleotide synthesis de novo | [27] |
| BRPI/pLAC4-SEF1 | BRPI with overexpression of *SEF1* gene under control of lactose-inducible promoter *LAC4* gene | Present study |
| BRPI/RIB6 | BRPI with expression of *RIB6* gene | [29] |
| L2/pTEF1-GND1 | L2 with expression of *C. famata GND1* gene encoding 6-phosphogluconate dehydrogenase under control of *C. famata TEF1* | Present study |
| AF-4/pTEF1-GND1 | AF-4 with expression of *C. famata GND1* gene encoding 6-phosphogluconate dehydrogenase under control of *C. famata TEF1* | Present study |
| BRP/pTEF1-GND1 | BRP with expression of *C. famata GND1* gene encoding 6-phosphogluconate dehydrogenase under control of *C. famata TEF1* | Present study |

Additional file 1 **Table 2.** Relative expression levels of *LAC4* gene in AF-4 strain cultivated in medium with different carbon source and *GND1* gene in the *GND1* overexpressing strains L2/pTEF1-GND1, AF-4/pTEF1-GND1 and BRP/pTEF1-GND1 versus the recipient strains L2, AF-4 and BRP. The relative expression levels were obtained via the comparative Ct method for quantification of the ΔΔC_t_ values. Error bars indicate standard deviations calculated from at least two independent experiments performed in triplicates. Strains were cultivated on YNB medium supplemented with yeast extract and 2% glucose, lactose or whey at 28°C, 220 rpm

| **ΔΔCt** | ***LAC4*** | ***GND1*** |
| --- | --- | --- |
| AF-4 (glucose) / AF-4 glucose) | 1.00 | **-** |
| AF-4 (lactose) / AF-4 (glucose) | 1.74 ± 0.056 | **-** |
| AF-4 (whey) / AF-4 (glucose) | 1.84 ± 0.159 | **-** |
| L2/pTEF1-GND1 / L2 | - | 1.58 ± 0.16 |
| AF-4/pTEF1-GND1 / AF-4 | - | 11.13 ± 0.35 |
| BRP/pTEF1-GND1 / BRP | - | 3.74 ± 1.18 |

|  |
| --- |
| Additional file 1 **Figure 1.** **(A)** Linear scheme of plasmid pNTC/pLAC4_cf-SEF1_cf. Promotor of the *LAC4* gene of *C. famata* is indicated as light gray box; *SEF1 Cf* with own terminator – as open box; selective marker *NTC* – as black box. **(B)** PCR verification of BRP/RFE1/pLAC4-SEF1 and BRPI/pLAC4-SEF1 strains with pair of primers Ko1068/OL23, which amplify 1722 bp fragment (positive and negative controls indicated as + and − ; 1-4 - BRP/RFE1/pLAC4-SEF1; 5-9 - BRPI/pLAC4-SEF1 and L - 1 kb ladder). Strains 1 and 9 were used in further experiments. |

|  |
| --- |
| Additional file 1 **Figure 2.** **(A)** Linear scheme of plasmid pGND1. The *TEF1* promoter from *C. famata* and *TEF1* terminator from *D. hansenii* are indicated as a light grey boxes; *GND1* gene - square grid box; selective marker gene *ble* under control of *TEF1* promoter from *C. famata* conferring resistance to phleomycin is indicated as grey box. **(B)** PCR verification of L2/pTEF1-GND1, AF-4/pTEF1-GND1 and BRP/pTEF1-GND1 strains with pair of primers Ko1056/Ko1059, which amplify 1188 bp fragment (positive and negative controls indicated as + and − ; 1-2 - L2/pTEF1-GND1; 3-4 - AF-4/pTEF1-GND1 and 5-7 - BRP/pTEF1-GND1 and L - 1 kb ladder). Strains 1, 3 and 5 were used in further experiments. |
